# Effect of an Experimental Direct Pulp-capping Material on the Properties and Osteogenic Differentiation of Human Dental Pulp Stem Cells

**DOI:** 10.1038/srep34713

**Published:** 2016-10-04

**Authors:** Fan Yu, Yan Dong, Yan-wei Yang, Ping-ting Lin, Hao-han Yu, Xiang Sun, Xue-fei Sun, Huan Zhou, Li Huang, Ji-hua Chen

**Affiliations:** 1 State Key Laboratory of Military Stomatology & National Clinical Research Centre for Oral Diseases & Shaanxi key Laboratory of Stomatology, Department of Prosthodontics, School of Stomatology, Fourth Military Medical University, Xi’an, 710032, P. R. China; 2 Department of Stomatology, Lanzhou General Hospital, Lanzhou Command of PLA, Lanzhou, 730050, P. R. China; 3 State Key Laboratory of Military Stomatology & National Clinical Research Centre for Oral Diseases & Shaanxi International Joint Research Center for Oral Diseases, Department of Operative Dentistry & Endodontics, Fourth Military Medical University, Xi’an, Shaanxi, 710032, P. R. China; 4 State Key Laboratory of Military Stomatology & National Clinical Research Centre for Oral Diseases & Shaanxi International Joint Research Center for Oral Diseases, Department of General Dentistry and Emergency, School of Stomatology, Fourth Military Medical University, Xi’an, Shaanxi, 710032, P. R. China

## Abstract

Effective pulp-capping materials must have antibacterial properties and induce dentin bridge formation; however, many current materials do not satisfy clinical requirements. Accordingly, the effects of an experiment pulp-capping material (Exp) composed of an antibacterial resin monomer (MAE-DB) and Portland cement (PC) on the viability, adhesion, migration, and differentiation of human dental pulp stem cells (hDPSCs) were examined. Based on a Cell Counting Kit-8 assay, hDPSCs exposed to Exp extracts showed limited viability at 24 and 48 h, but displayed comparable viability to the control at 72 h. hDPSC treatment with Exp extracts enhanced cellular adhesion and migration according to *in vitro* scratch wound healing and Transwell migration assays. Exp significantly upregulated the expression of osteogenesis-related genes. The hDPSCs cultured with Exp exhibited higher ALP activity and calcium deposition *in vitro* compared with the control group. The novel material showed comparable cytocompatibility to control cells and promoted the adhesion, migration, and osteogenic differentiation of hDPSCs, indicating excellent biocompatibility. This new direct pulp-capping material containing MAE-DB and PC shows promise as a potential alternative to conventional materials for direct pulp capping.

Dental pulp is occasionally exposed by dental caries, trauma, or iatrogenic injury in clinical practice. Untreated pulp exposure can cause pulp necrosis in the case of bacterial infections, and can undermine the integrity of the tooth structure. Additionally, dental tissues under stress are associated with a high risk of tooth fracture. Pulp capping, including direct and indirect pulp capping, in which biocompatible and bioregenerative dental materials are used to seal the pulpal wound, facilitates the formation of a dentin bridge and maximises pulp preservation, while offering an alternative to root canal therapy^1^,^2^. A number of pulp-capping materials have been introduced. Historically, calcium hydroxide was the standard direct pulp-capping material because it facilitates the formation of a dentinal bridge over the pulp wound area^3^. However, it has many known disadvantages, such as a high solubility, a lack of adhesive qualities, degradation after acid etching, and the presence of tunnels in the dentinal bridge, which allow the pulp to become infected or necrotic over time; thus, it cannot prevent the pulp from continued bacterial penetration owing to its poor sealing ability^4^,^5^.

Calcium silicate-based cements, such as mineral trioxide aggregate (MTA), Biodentine, and Portland cement (PC), have been studied for applications to direct pulp capping^2^,^6^,^7^. MTA is an alternative to Ca(OH)_2_ that achieves the same results in clinical procedures and can induce a significantly greater frequency of dentinal bridge formation, bridges with greater mean thicknesses and less pulp inflammation^8^,^9^,^10^. However, MTA has some disadvantages, such as a prolonged setting time, high cost, potential for discolouration, poor handling characteristics, and inadequate antibacterial effects^11^,^12^. Many studies have focused on PC, which is compositionally similar to MTA, but less expensive. Several studies have suggested that PC is biocompatible and can be used as a safe pulp-capping material, and many PC-based materials have been investigated to meet clinical requirements^13^,^14^.

Although contemporary pulp-capping materials have demonstrated various benefits, microorganisms are one of the main reasons for failure^15^. Accordingly, it is necessary to develop a material that can effectively limit bacterial infection and induce dentin bridge formation^11^. In our previous study, we synthesised a resin-based direct pulp-capping material that contained the quaternary ammonium salt monomer MAE-DB and PC^16^. This novel material showed constant contact inhibition against *Streptococcus mutans in vitro*, even after 6 months in water^16^. We speculated that the Ca and Si from PC might confer favourable properties and bioactivity for pulp healing, similar to MTA^17^,^18^,^19^.

The cell viability, adhesion, migration, and differentiation of human dental pulp stem cells (hDPSCs) have been implicated in the healing response to pulp injury process^20^. Thus, in the present study, we investigated the effect of the experimental direct pulp-capping material containing MAE-DB and PC (Exp) on hDPSCs in terms of cell viability, migration, adhesion, and osteogenic differentiation.

## Methods

### Reagents

The following reagents were used in the study: 2-hydroxyethylmethacrylate (HEMA, Sigma–Aldrich, St. Louis, MO, USA), bisphenol glycerolate dimethacrylate (BisGMA; Esstech, Essington, PA, USA), triethylene glycol dimethacrylate (TEGDMA, Esstech), photoinitiator campho9rquinone (CQ, Sigma-Aldrich), ethyl 4-(dimethylamino)benzoate (EDMAB, Sigma-Aldrich), and white PC (P. W. Grade 52.5) provided by the Aalborg Portland Group (Anqing, China). The QAS antibacterial monomer (MAE-DB) was developed by our team.

### Sample preparation

The new resin-based composite containing MAE-DB and PC was fabricated according to the methods described in our previous study^16^. Briefly, a synthesised resin matrix containing HEMA-BisGMA-TEGDMA (mass ratio, 4:3:1) with MAE-DB at 5 wt% was mixed with white PC (P. W. 52.5, Aalborg) at a PC: HEMA-BisGMA-TEGDMA resin mass ratio of 2:1. The resins were photo-cured for 60 s on each side with a light activation unit (QHL75; Dentsply, Tulsa, OK, USA) in a 24-well plate. A cured resin matrix without PC was fabricated for the cell viability assay. White ProRoot MTA (Dentsply) (hereafter referred to as MTA) was used as a commercially available material for comparison; it was prepared according to manufacturer’s instructions on the bottom of a 24-well plate and left to set for 48 h at 37 °C in a humidified 5% CO_2_, 95% air atmosphere. All of the prepared disks were sterilised with ethylene oxide before the subsequent experiments.

The sample elutes were used for the cell adhesion assay and cell migration assay. The ratio between the surface of the samples and the volume of the medium (α-MEM containing 5% foetal bovine serum [FBS]) was 0.5 cm^2^/mL, according to ISO standards (10993-5). After incubation at 37 °C for 24 h, the resulting supernatant was sterilised using a 0.22-μm filter before use with the cell cultures and was changed every three days.

### Isolation and culture of hDPSCs

Human third molars or premolars extracted from young orthodontic patients (18–19 years old) after obtaining informed consent were collected for the further study. All the experimental protocols were reviewed and approved by the Institutional Review Board of Stomatological Hospital of Fourth Military Medical University (approval number: IRB-REV-2015036; Supplementary information). Methods were carried out in accordance with relevant guidelines and regulations. Briefly, freshly extracted teeth were washed with phosphate-buffered saline (PBS) supplemented with antibiotics. Dental pulp tissues were separated and minced into 1 mm^2^ fragments, followed by digestion with a solution of 3 mg/mL type I collagenase with 4 mg/mL dispase (Sigma) for 45–60 min at 37 °C. Single-cell suspensions were obtained by passing the cells through a 70-μm cell strainer followed by incubation in α-MEM supplemented with 20% FBS (HyClone, Logan, UT, USA), 100 units/mL penicillin G, 100 mg/mL streptomycin, and 50 mg/mL ascorbic acid (Sigma) at 37 °C in 5% CO_2_. Flow cytometry was used to characterise hDPSCs. The cells between the third and fifth passages were used in the subsequent analysis.

### Flow cytometry assay

The cultured cells were identified based on the surface antigens of hDPSCs using a flow cytometry method. Cells were trypsinised and incubated in PBS containing 0.1% FBS for 45 min with fluorescein-conjugated monoclonal antibodies against CD29, CD34, CD45, CD90, and CD105 (BD Biosciences, San Jose, CA, USA). The flow cytometry test was performed using a flow cytometer (FACSCalibur; BD Biosciences).

### Cell viability assay

Cell viability was measured using the Cell Counting Kit-8 (7Sea Biotech, Shanghai, China) according to the manufacturer’s protocol. Briefly, hDPSCs were plated at a density of 1 × 10^4^ cells per well and a volume of 100 μL in 96-well plates. The cells were starved for 24 h with serum-free medium at 37 °C in a humidified 5% CO_2_ atmosphere before the test. Then the serum-free medium was replaced with different material elutes. Cells cultured in α-MEM medium containing 10% FBS served as a blank control. At each time point (24, 48, and 72 h after seeding cells), 10 μL of CCK8 assay solution was added to each well and incubated for 4 h at 37 °C. Cell number and viability were calculated by measuring absorbance at a wavelength of 450 nm on a multi-plate reader (BIO-TEK, Winooski, VT, USA). The experiments were performed in triplicate.

### Adhesion assay

Type I collagen (40 mg/L in PBS; Corning, Discovery Labware, Inc., Bedford, MA, USA) was added to 24-well plates and placed at 4 °C overnight. Subsequently, unbound collagen was removed using PBS and the nonspecific binding sites were blocked with 1% bovine serum albumin (BSA) at 37 °C in a 5% CO_2_ atmosphere for 1 h. hDPSCs (1 × 10^4^ cells/mL) were resuspended in 100 μL of elute prepared with serum-free α-MEM and were seeded on the wells. After incubation at 37 °C in a 5% CO_2_ atmosphere for 1 h, non-adherent cells were rinsed with PBS and adherent cells were fixed in 4% formaldehyde for 10 min and stained with 0.5% toluidine blue for 10 min. An inverted phase contrast microscope (Olympus, Tokyo, Japan) was used to obtain images and the wells were then washed with PBS and supplemented with 33% acetic acid (v/v). The optical density (OD) was determined using a microplate reader (BIO-TEK) at an absorbance of 595 nm.

### Migration assay

#### Transwell migration assay

Cell migration was assessed using a two-chamber Transwell system (8 mm pore size and 6.5 mm diameter). hDPSCs (1 × 10^4^ cells/mL) suspended in 100 μL of serum free α-MEM were planted in the top chamber of the Transwell, and 500 μL of the eluate prepared with serum-free α-MEM was added to the lower chambers. After incubation at 37 °C in 5% CO_2_ for 24 h, filters were removed with sterile tweezers and cells that did not migrate through the filter were gently wiped with a cotton swab. Migrating cells beneath the filter were fixed with 4% paraformaldehyde for 15 min and stained with crystal violet for 20 min. The filters were observed using an inverted microscope (Olympus). The numbers of the migrating cells in each well were counted in 6 random microscopic fields per filter at 200× magnification. The experiments were performed in triplicate independently.

#### Wound healing assay

Cell migration was also assessed using an *in vitro* scratch wound healing assay. hDPSCs were cultured with α-MEM supplement with 10% FBS until 100% confluence in 6-well plates. Then, the cells were starved overnight with serum-free α-MEM and a scratch was made using a sterile 1-mL pipette tip. The cell debris was removed with PBS and cells were cultured with elutes for 24 h. An inverted microscope was used to observe cell migration.

### *In vitro* osteogenic differentiation assay

hDPSCs were co-cultured with various material extracts and serum-free α-MEM. Normal culture medium containing α-MEM supplemented with 10% FBS was used as blank control medium (Control/N). Cells cultured with osteogenic differentiation medium were used as a positive control (Control/M). Control/M consisted of the aforementioned normal culture medium supplemented with 50 mg/mL ascorbic acid, 10 mmol/L beta-glycerophosphate, and 10 nmol/L dexamethasone (all from Sigma). The effects of MTA and PC on hDPSC osteogenic differentiation were evaluated.

#### Alizarin Red staining and quantification

Approximately 1 × 10^5^ hDPSCs were cultured with the elutes supplemented with serum-free α-MEM in 6-well plates for 2 weeks. The cells were washed with PBS and fixed with dehydrated ethanol for 20 min. Cells were stained using the Alizarin Red S Kit (Leagene Biotechnology, Beijing, China) according to the manufacturer’s instructions for 20 min and then washed with H_2_O 5 times. To quantify Alizarin Red staining, the stained cells were incubated in 10% acetic acid for 30 min and absorbance was measured at 405 nm with a microplate reader.

#### Alkaline phosphatase staining and quantification

For alkaline phosphatase (ALP) staining, approximately 1 × 10^5^ hDPSCs were seeded in 6-well plates. After reaching 80% confluence, the cells were divided into five groups (MTA, PC, Exp, Control/N, and Control/M). After 9 days of cultivation, wells were washed twice with PBS and fixed with 4% polyoxymethylene for 30 min at room temperature. An ALP Staining Kit (Jiancheng Bioengineering Institute, Jiangsu, China) was used according to the manufacturer’s instructions. After staining for 30 min at room temperature, cells were observed under a light microscope. To quantify ALP activity, hDPSCs were plated in 96-well plates at a density of 1 × 10^4^ hDPSCs/well and incubated overnight in 5% CO_2_ at 37 °C. After incubation in media without FBS for 24 h, the samples were then divided into the 5 groups described above; each group included 5 wells and media was changed every 3 days. On days 3, 5, 7, and 9, the ALP activity of each well was assayed with an ALP Kit (Jiancheng Bioengineering Institute) according to the manufacturer’s instructions. Absorbance was measured at 520 nm with a microplate reader.

#### Quantitative real-time polymerase chain reaction

Osteogenic potential was examined using the bone-associated markers ALP, osteonectin (ON), osteocalcin (OCN), dentin matrix protein 1 (DMP-1), and dentin sialophosphoprotein (DSPP) using qRT-PCR. hDPSCs (1 × 10^5^) were seeded in 6-well plates and divided into five groups. After incubation for 2 weeks, the total RNA was extracted and reverse transcribed into complementary DNA with reverse transcriptase using the TaKaRa MiniBEST Universal RNA Extraction Kit and PrimeScript™ RT Master Mix (Perfect Real Time, Takara, Otsu, Japan) according to the manufacturers’ protocols. Subsequently, PCR amplification was performed with SYBR^®^ Premix Ex Taq™ (Tli RNaseH Plus) (Takara) using the ABI 7500 Thermal Cycler (Applied Biosystems, Foster City, CA, USA). The PCR primers are shown in Table 1. The gene expression levels were normalised to the β-actin mRNA level and averaged from triplicate samples.

### Statistical analysis

The data are expressed as means ± standard deviations of three independent experiments performed in triplicate. We preformed normal distribution test and homogeneity test for error variance and the results showed that the distribution of the dates in this study were all alone to normal distribution after test of normality. The p values of homogeneity test are all greater than 0.05. Thus One-way ANOVA and LSD post-hoc tests were used to determine differences among groups using SPSS 19.0. Statistically significant differences were accepted at P < 0.05.

## Results

### Characterisation of hDPSCs

**Figure 1:**
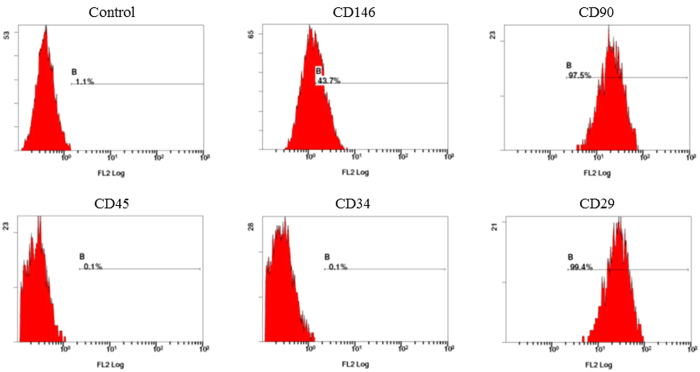
Flow cytometry of mesenchymal-related antigens in human dental pulp stem cells (hDPSCs). Representative diagrams are given for the negative control, CD146, CD90, CD45, CD34, and CD29 expression.

Based on flow cytometry, cells exhibited high expression of mesenchymal surface molecular markers (CD29, 99.4%; CD146, 43.7%; CD90, 97.5%) and low expression of surface makers for hematopoietic system-derived cells (CD34, 0.1%; CD45, 0.1%) (Fig. 1).

### Cell viability

**Figure 2:**
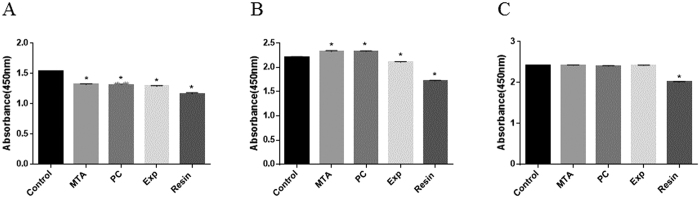
Results of cell viability assays after hDPSCs were exposed to various material extracts at 24 h (**A**), 48 h (**B**), and 72 h (**C**). The viability of cells under treatments with different material extracts was investigated using the Cell Counting Kit-8. Data are shown as means ± standard deviation of three independent experiments. *P < 0.05 represents a significant change compared with the control.

Using the CCK-8 assay, the viability of hDPSCs exposed to various material extracts was examined at 24 h, 48 h, and 72 h. As shown in Fig. 2, the viabilities of cells exposed to MTA, PC, Exp, and Resin were significantly lower than that of the negative control group (P < 0.001) at 24 h, and no significant differences were found among the MTA, PC, and Exp groups (P > 0.05). After 48 h of incubation, cells exposed to Exp showed lower viability than the control group (P < 0.001), while viability was higher in the MTA and PC groups than the control (P < 0.001). The Resin group showed significantly lower cell viability than all other groups. (P < 0.001). After 72 h, there were no significant differences among the MTA, PC, Exp, and control groups (P > 0.05), all of which had higher viabilities than that of the Resin group (P < 0.001).

### Cell Adhesion

**Figure 3:**
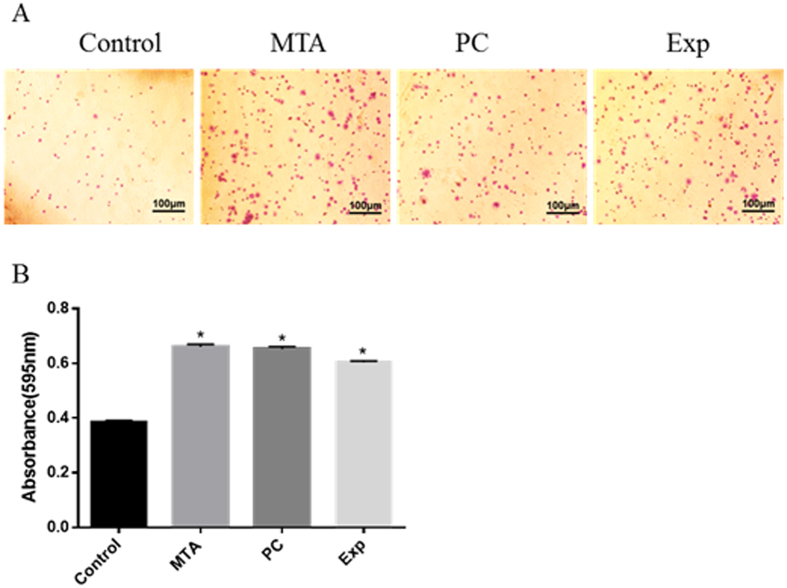
The effect of material extracts on adhesion ability in hDPSCs. Cells were incubated with various material extracts for 1 h. Adherent cells were fixed and stained. The coloured solution was quantified at 595 nm on a microplate reader. (**A**) Representative diagrams for stained cells. (**B**) Quantification of the adherent cells. Data are presented as means ± standard deviation and measurements were performed in triplicate, with results summarised as the mean for each experiment. *P < 0.05 represents a significant change compared with the control.

As shown in Fig. 3, considerably more adherent cells were detected for the Exp group than the control. There were no significant differences among the MTA, PC, and Exp groups (P > 0.05).

### Cell Migration

**Figure 4:**
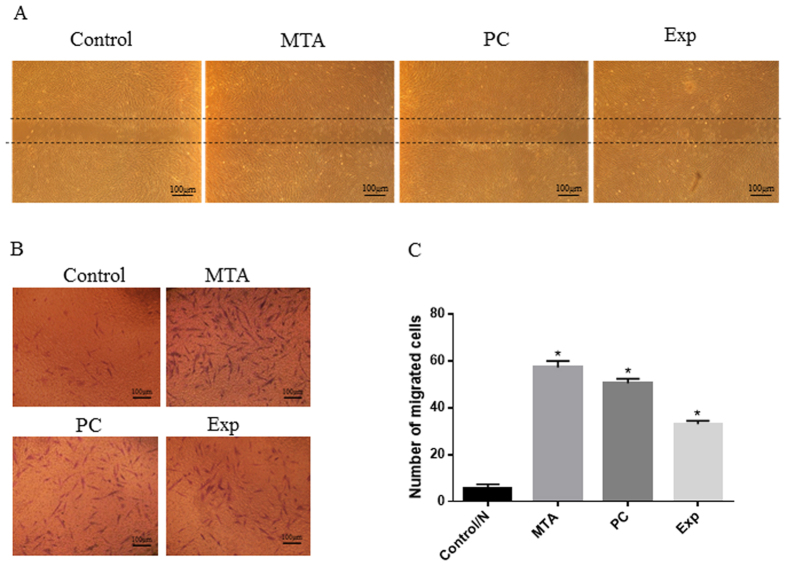
Effects of material extracts on migration in hDPSCs. (**A**) Wound-healing assay. Cells were incubated with different material extracts for 24 h. Microphotographs of the scratches were obtained at 24-h post-wounding. (**B**) Cell migration assays were performed using a two-chamber Transwell system. Cells were treated with different material extracts for 24 h, and the migrated cells were fixed and stained. Representative photos of migrated hDPSCs were observed under a phase-contrast microscope. (**C**) Quantification of migrated cells. Data are presented as means ± standard deviation of three independent experiments. *P < 0.05 represents a significant change compared with the control.

In order to determine the effect of the extracts on the motility of hDPSCs, a wound-healing assay and a two-chamber Transwell assay were performed. As shown in Fig. 4A, Exp shortened the migration distance compared with the control group. Based on the Transwell assay, the numbers of migrated cells in the MTA, Exp, and PC groups were significantly higher than that of the control (P < 0.001), while no detectable differences were observed among the MTA, PC, and Exp groups (P > 0.05) (Fig. 4B,C). These results indicated that Exp had a positive effect on the migration ability of hDPSCs.

### *In vitro* osteogenic differentiation assay

#### Alizarin Red staining and quantification

**Figure 5:**
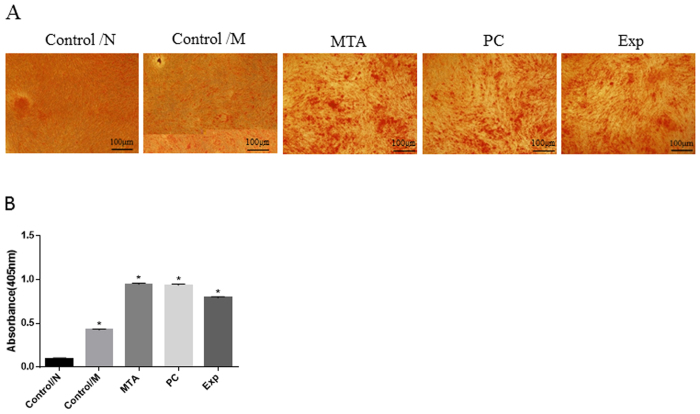
Alizarin Red staining and quantification. hDPSCs were co-cultured with different material extracts supplemented with serum-free α-MEM for 2 weeks. Normal culture medium containing α-MEM supplemented with 10% foetal bovine serum was used as blank control medium (Control/N). Cells cultured with osteogenic differentiation medium were used as positive controls (Control/M). (**A**) Mineralised nodules formed by differentiated cells after incubation were stained with Alizarin Red S. (**B**) Quantitative measurements of Alizarin Red S staining of hDPSCs. The results are expressed as means ± standard deviation and *P < 0.05 represents a significant difference compared with the control/N group.

Mineral nodule formation is often used to assay the differentiation of hDPSCs and Alizarin Red staining is an indicator of mineralized-like nodules. Figure 5 shows representative images of calcium deposits produced by hDPSCs in each group. The least calcium deposition was observed for hDPSCs in the Control/N group (P < 0.001). Compared with the Control/M group, Exp significantly promoted calcium deposition in hDPSCs (P < 0.001), and more calcium deposition was detected for the MTA and PC groups compared with the other groups (P < 0.001).

#### Alkaline phosphatase staining and quantification

**Figure 6:**
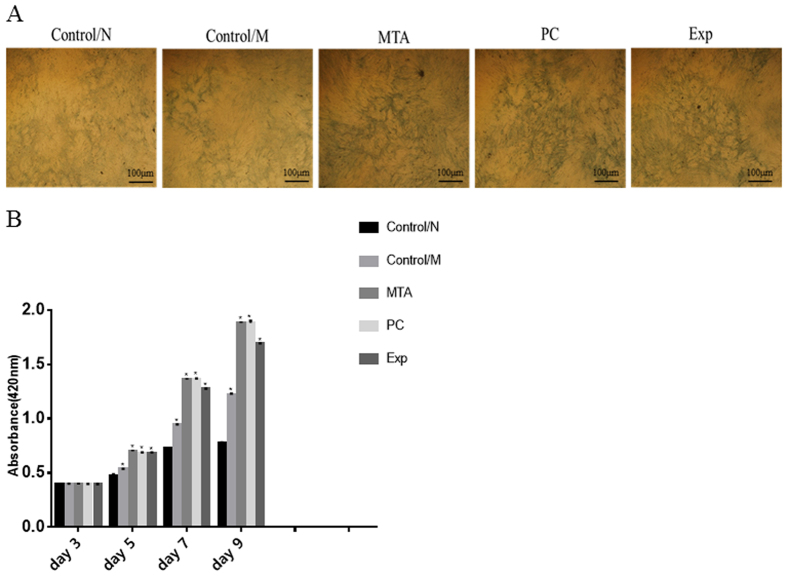
Alkaline phosphatase (ALP) staining and quantification. Cells were co-cultured with different material extracts supplemented with serum-free α-MEM. Normal culture medium containing α-MEM supplemented with 10% FBS was used as blank control medium (Control/N). Cells cultured with osteogenic differentiation medium were used as positive controls (Control/M). ALP staining after 9 days of cultivation was performed using an ALP Staining Kit (**A**). ALP activity was evaluated at 3 days, 5 days, 7 days, and 9 days using an ALP Kit (**B**). The results are expressed as means ± standard deviation and *P < 0.05 represents a significant difference compared with the control/N group.

Results of ALP staining are shown in Fig. 6. After 2 weeks of mineralization induction, compared with the control/M group and the control/N group, Exp, MTA, and PC induced positive ALP staining (Fig. 6A). The ALP activity of hDPSCs on days 3, 5, 7, and 9 is indicated in Fig. 6B. The ALP activity of all groups increased until day 9. Compared with the control/M and control/N groups, treatment with Exp markedly increased ALP activity (P < 0.001), and treatment with MTA and PC had the greatest effect on ALP activity at day 9 (P < 0.001).

#### Quantitative real-time polymerase chain reaction

**Figure 7:**
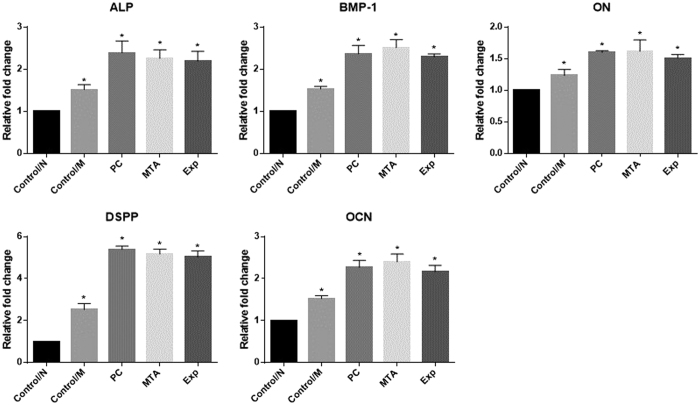
The effects of material extracts on the expression of *ALP*, *BMP-1*, *ON*, *DSPP*, and *OCN* in hDPSCs. Cells were co-cultured with different material extracts supplemented with serum-free α-MEM for 2 weeks. Normal culture medium containing α-MEM supplemented with 10% FBS was used as blank control medium (Control/N). Cells cultured with osteogenic differentiation medium were used as positive controls (Control/M). *ALP*, *BMP-1*, *ON*, *DSPP*, and *OCN* mRNA expression levels in hDPSCs were determined by qRT-PCR. The results are expressed as means ± standard deviation and *P < 0.05 represents a significant difference compared with the control/N group.

Figure 7 summarises the expression levels of osteogenic differentiation markers in hDPSCs for each group, which were normalised to β-actin. After exposure to the five treatments for 14 days, the hDPSCs exposed to Exp exhibited significantly higher expression levels of *OCN*, *DSPP*, *DMP1*, *ON*, and *ALP* mRNA than the control/N and Control/M groups (P < 0.05), while no detectable differences were observed among the MTA, PC, and Exp groups (P > 0.05).

## Discussion

Direct pulp capping is defined as the capping of exposed vital pulp with a bioactive material to maintain dental pulp viability and facilitate the formation of reparative dentin. It offers an alternative conservative treatment approach to root canal therapy^2^. hDPSCs in dental pulp tissue show the capacity for self-renewal and multilineage differentiation, and are a source of pulp healing^21^,^22^,^23^. When exposed pulp is capped by biocompatible materials, hDPSCs proliferate, migrate to the injured site, and differentiate into odontoblast-like cells, which subsequently deposit reparative dentine^22^,^24^. In addition to appropriate physical properties, nontoxicity to pulp tissue, acceptable sealing ability, a short setting time, and excellent handling and antibacterial properties, an ideal pulp-capping material should also demonstrate the ability to stimulate and modulate the healing process induced by hDPSCs^25^. Although numerous materials have been developed, none fulfils these clinical demands. Exp was created in an attempt to combine the antibacterial properties of MAE-DB with the biological properties of PC. In the present study, its effects on the proliferation, adhesion, migration, and odontogenic differentiation of the hDPSCs were evaluated.

The influence of cytotoxic compounds from pulp-capping materials on cell viability and apoptosis is of great concern^26^,^27^. The preservation of pulp vitality following restorative intervention depends on the degree to which pulpal cell populations can survive^28^. Pulp-capping materials should either promote cell survival and proliferation or be biologically neutral^29^. In our study, cell viability was significantly reduced when cells were cultured with MTA, PC, and Exp compared with the control group for 24 h, but there were no significant differences among the MTA, PC, Exp, and control group at 72 h, indicating a comparatively good cytocompatibility. The cured resin without PC exhibited significant cytotoxicity at each time point. Resin monomers can induce cell death via an ROS pathway^30^,^31^, while MTA can promote cell proliferation at certain concentrations^32^. Thus, we speculated that the PC constituent in Exp may offset the negative effect induced by resin monomers leached from the material. This phenomenon may also be explained by the increase in resin components in the Resin than the Exp group.

The adhesion and migration behaviours of hDPSCs are fundamental during pulp healing. Accordingly, it is vital to clarify the changes in hDPSCs migration and adhesion with respect to dentin–pulp regeneration when different pulp-capping materials are used^33^,^34^. As shown in Figs 3 and 4, Exp promoted cellular migration and adhesion. Our findings are in agreement with previous studies showing that MTA could accelerate the migration and adhesion of dental pulp-derived cells^35^,^36^. A migration assay also provided the first *in vitro* evidence of a similar migration effect of PC on hDPSCs, but Vincenzo *et al.*^37^ found that MTA enhances HMSC migration significantly more than PC. Interestingly, in our study, the number of migrated cells in the Exp group was significantly higher than that of the control group, but less than those of the MTA and PC groups. We speculated that the reduced biocompatible could be attributed to the HEMA(2-hydroxyethyl methacrylate) in Exp, which, according to Williams *et al.*^38^, inhibits the migration of DPSCs.

Bioactive materials used for pulp tissue healing should enhance the dentinogenic potential of pulp stem cells. In the present study, odontoblastic differentiation-related makers (*ALP*, *DMP-1*, *DSPP*, *OCN*, and *ON*) were tracked by qRT-PCR, and intracellular ALP enzyme activity and matrix mineralization were detected using ALP and Alizarin Red staining. ALP is a marker of early odontoblast differentiation and plays a key role in reparative dentin mineralization^39^. DSPP, composed of two odontoblast-specific proteins, dentin sialoprotein and dentin phosphoprotein, is related to dentinogenesis^40^. DMP1 regulates collagen matrix organization and promotes dentin matrix mineralization, similar to DSPP^41^,^42^. OCN and ON are also thought to be terminal indicators of dentin regeneration^43^,^44^,^45^. The increased and earlier upregulation of these genes suggest that Exp could promote hDPSCs to odontogenic differentiation. These findings are consistent with the increase in calcified nodules in Exp detected by Alizarin Red staining compared with the control group. Thus, we can conclude that Exp enhances the odontogenic potential of hDPSCs, similar to MTA and PC^46^,^47^.

Taken together, the results of this study indicate that Exp supports tissue regeneration via the promotion of human pulp stem cell adhesion, migration, and differentiation. These result reflect the similarity in the compositions of Exp and MTA based on PC, which is biocompatible in cultures of several cell lines^48^,^49^,^50^,^51^. Calcium silicate-based materials, include MTA, PC, and Biodentine, were believed to release Ca ions, which can trigger the recruitment and proliferation of undifferentiated cells from the pulp^52^ and activate stem cells^53^. However, these materials lack effective antimicrobial properties^54^. Torabinejad *et al.*^55^ reported that Loma Linda MTA does not exert an inhibitory effect against *Enterococcus faecalis, Staphylococcus aureus*, or *Fusobacterium nucleatum*. In a study of GMTA mixed with sterile water, no anti-bacterial effect against *E. faecalis* was observed^55^. Another study reported only bacteriostatic effects of MTA against *E. faecalis* using a direct contact testing protocol^56^. However, this activity of MTA is limited against some facultative bacteria and has no effect on strictly anaerobic bacteria^55^.

Recontamination by the restoration of microleakages and residual bacteria in dentin tubes may have adverse effects on pulp healing. The presence of bacteria and their byproducts stimulate pulpal inflammatory activity and reduce the area of dentin bridge formation, irrespective of the material used for pulp capping, and subsequently lead to the failure of pulp capping^57^,^58^, even when a dentine bridge forms^59^. Cox *et al.*^60^ have shown that pulp healing is more highly dependent on the capacity of the capping material to prevent bacterial microleakage than on the specific properties of the material itself. In our previous experiments, Exp released comparable amounts of Ca ions as MTA and exhibited effective antibacterial activity throughout the experimental period, which promotes pulp healing.

In summary, the experimental direct pulp-capping material containing MAE-DB and PC showed comparable cytocompatibility on hDPSCs and induced adhesion, migration, and osteogenic differentiation *in vitro*. Based on its favourable bioactivities and effective antibacterial characteristics, this new direct pulp-capping material shows promise as used for vital pulp therapy. *In vivo* examinations of its effect on dentine bridge formation are in progress.

## Additional Information

**How to cite this article**: Yu, F. *et al.* Effect of an Experimental Direct Pulp-capping Material on the Properties and Osteogenic Differentiation of Human Dental Pulp Stem Cells. *Sci. Rep.* **6**, 34713; doi: 10.1038/srep34713 (2016).

## Figures and Tables

**Table 1:** Primer sequences used for qRT-PCR analysis.

Gene	Code	Forward primer sequences	Reverse primer sequences
ALP	NM 000478	CATGCTGAGTGACACAGACAAGAA	ACAGCAGACTGCGCCTGGTA
ON	MM 182120	GAAGGCCAAAATCAAGAGTGAGA	AAAAGGGGAGGGTGAAGAAAAG
OCN	NM 199173	GACGAGTTGGCTGACCACA	CAAGGGGAAGAGGAAAGAAGG
DMP-1	NM 004407	ACTGTGGAGTGACACCAGAACACA	AGCTGCAAAGTTATCATGCAGATCC
DSPP	NM 014208	GCATTTGGGCAGTAGCATGG	CTGACACATTTGATCTTGCTAGGAG
β-actin	NM 001101.3	CATGGATGATGATATCGCCGCG	ACATGATCTGGGTCATCTTCTCG
